# Carriage and colonization of *C*. *difficile* in preterm neonates: A longitudinal prospective study

**DOI:** 10.1371/journal.pone.0212568

**Published:** 2019-02-20

**Authors:** Laurent Ferraris, Jeanne Couturier, Catherine Eckert, Johanne Delannoy, Frédéric Barbut, Marie-José Butel, Julio Aires

**Affiliations:** 1 EA 4065, Faculty of Pharmacy, Paris Descartes University, Hospital University Department Risks in pregnancy, Sorbonne Paris Cité, Paris, France; 2 French National Reference Laboratory for *C*. *difficile*, CHU Saint-Antoine, Paris, France; 3 Department of Bacteriology, AP-HP, GH Est Parisien, Paris, France; University of Arizona, UNITED STATES

## Abstract

**Background:**

Premature neonates (PN) present multiple risk factors for high frequencies and high levels of colonization by *C*. *difficile*, yet data is missing about this specific pediatric population. Here, we investigated PN *C*. *difficile* carriage and colonization dynamics, analyzed the impact of perinatal determinants on colonization, and characterized the isolates.

**Methods:**

A one year longitudinal monocentric prospective cohort study was performed on 121 PN. *C*. *difficile* strains isolated from fecal samples on selective medium were identified and characterized by PCR (*tpi* housekeeping gene; *tcdA* and *tcdB*, and binary toxin genes), capillary gel-based electrophoresis PCR-ribotyping, and Multi-Locus Variable-number tandem-repeat Analysis (MLVA).

**Results:**

Of the 379 samples analyzed, 199 (52%) were *C*. *difficile* culture positive with the mean levels of *C*. *difficile* colonization decreasing significantly (P = .027) over time. During hospitalization, *C*. *difficile* colonization frequency increased up to 61% with 95% of the strains belonging to both non-toxigenic PCR-ribotypes (RTs) FR082 (35%) and 032 (60%). After hospital discharge, if a higher diversity in RTs was observed, RTs FR082 and 032 remained predominant (respectively 40% and 28%). MLVA showed clonal relationship within each FR082 and 032 RTs. Ten toxigenic strains (5%) were isolated, all *tcdA*^+^/*tcd*B^+^ except for one *tcdA*^-^/*tcd*B^+^, and all being acquired after hospitalization. At 1 week, the only factors found to be linked with a higher frequency of *C*. *difficile* colonization were a higher gestational age (P = 0.006) and a higher birth weight (P = 0.016).

**Conclusion:**

The dynamics of *C*. *difficile* colonization in PN followed a specific pattern. *C*. *difficile* colonization rapidly occurred after birth with a low diversity of non-toxigenic RTs. After hospitalization, non-toxigenic RTs diversity increased. Sporadic carriage of toxigenic strains was observed after hospitalization.

## Introduction

*Clostridium difficile* is a gram-positive, anaerobic spore-forming bacillus that was first isolated as a commensal bacterium in healthy neonates [1]. Nowadays, in developed countries, *C*. *difficile* is recognized as the major cause of antibiotic-associated diarrhea and colitis in adults and is associated with a substantial morbidity and mortality [2]. Its major virulence factors include toxins A (TcdA), B (TcdB), and binary toxin (Cdt) in some strains [2]. Worldwide hyper-virulent strains (e.g. 027 or 078) have become a significant cause of nosocomial infections and outbreaks of *C*. *difficile* severe infections [3;4]. So far, non-toxigenic *C*. *difficile* strains are considered non-pathogenic.

Several reports have documented a change in the epidemiology of *C*. *difficile* infections (CDI) with an increasing incidence of community-associated CDI in both adults [5] and pediatric populations [6;7]. Moreover, CDI are occurring among populations without traditional risk factors [8;9]. Therefore, there is an increasing interest in establishing whether asymptomatic *C*. *difficile* colonized populations may represent a potential reservoir for *C*. *difficile* transmission and spread in both health-care and community environments. This will help guide strategies to prevent community-associated CDI.

*C*. *difficile* carriage can occur in healthy adults, but rates increase to 20–50% in hospitalized patients or in those in long-term facilities [10]. In children, up to 70% of infants less than 1 year old have been shown to be asymptomatic carriers of toxigenic and/or non-toxigenic *C*. *difficile* strains, with colonization falling to adult levels by age of 2 years [10;11]. Data regarding *C*. *difficile* colonization in preterm neonates (PN) are scarce although they have multiple risk factors for high frequencies and high levels of colonization by *C*. *difficile*. Indeed, PN are characterized by long hospitalization periods, frequent administration of antibiotics to prevent early-onset infections, and lack of protective intestinal microbiota [12]. Besides, *C*. *difficile* was shown to be one of the three most frequent clostridia species isolated from PN fecal samples [13]. The aims of this study were to determine the frequency of asymptomatic *C*. *difficile* colonization and its dynamics in PN through a one year longitudinal study, to analyze the perinatal determinants of *C*. *difficile* colonization, and to characterize the isolated strains.

## Materials and methods

### Study

**Fig 1:**
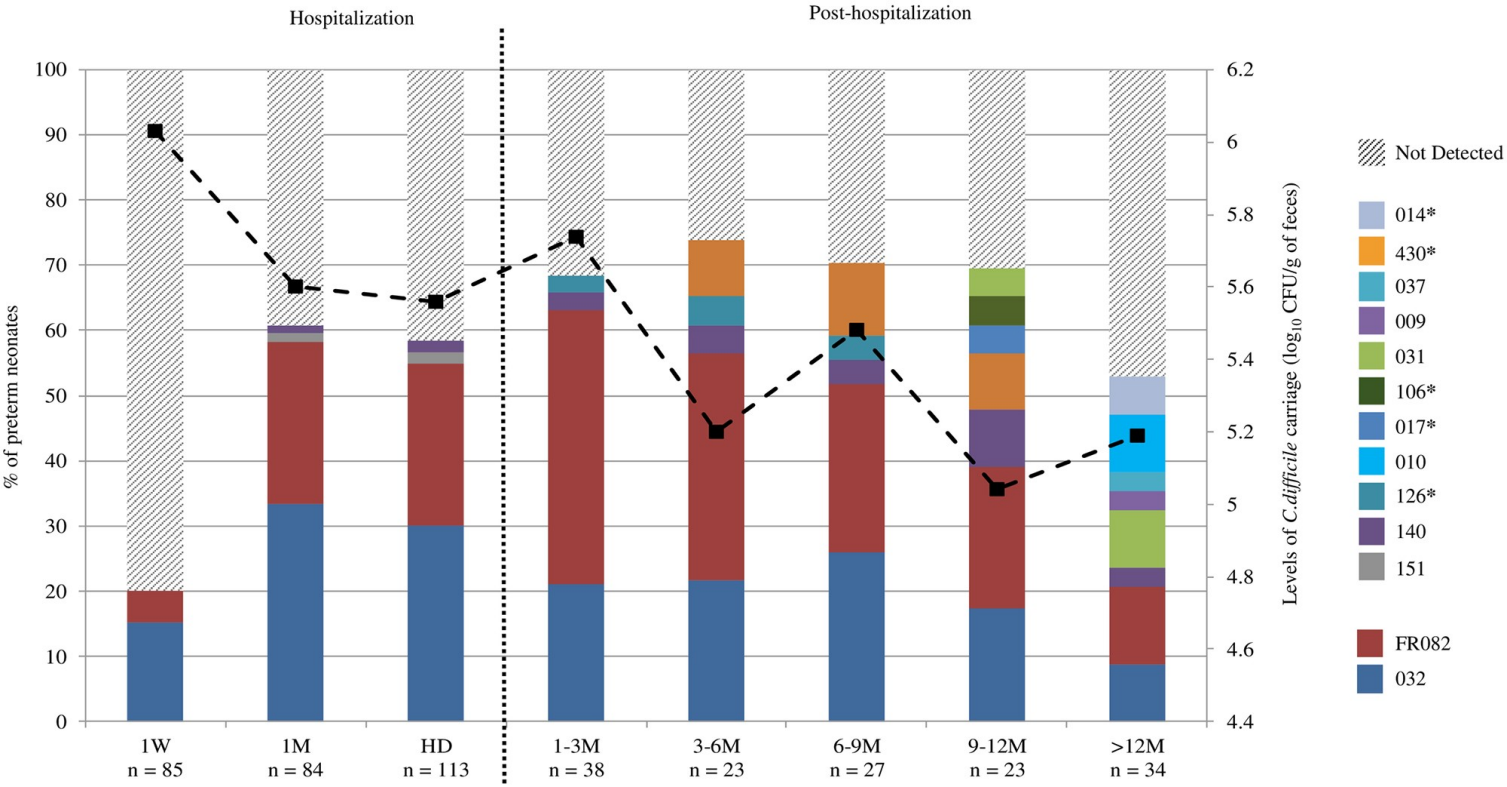
*C*. *difficile* colonization and PCR-ribotypes distribution during the hospitalization and post-hospitalization periods. The bars represent the percentage of the preterm neonates and the black dashed line represents the mean levels of *C*. *difficile* carriage. PCR-ribotypes references were either the standard nomenclature from the European collection (i.e. 032) when available or designated by the French National Reference Laboratory specific nomenclature (i.e. FR082). The fecal samples included for analysis during hospitalization were gathered as follows: 1 week (1W) (≤8 days (d)) (n = 85); 1M, (20 d-40 d) (n = 84); hospital discharge (HD) (4 d-149 d) (n = 113). Since the postnatal age of the PN at HD was depending on factors such birth gestational age and health status, of the 113 HD samples, 6 and 42 corresponded respectively to the 1W and 1M sampling time points. For the post-hospitalization period, samples analyzed were distributed as follows: 1–3 M (n = 38); 3–6 M (n = 23); 6–9 M (n = 27); 9–12 M (n = 23); > 12 M (maximum 487 d) (n = 34). Not detected: threshold <3.3 log_10_ CFU/g. *, toxigenic strains.

PN were enrolled and followed during a 12-month prospective study (2008–2009) in a French NICU (Saint Vincent de Paul, Paris, France). Fecal samples were collected during hospitalization at one week and one month of postnatal age, and at hospital discharge (HD). After HD, feces were collected throughout one year at various postnatal age, i.e.1 to 3 months (M), 3 to 6 M, 6 to 9 M, 9 to 12 M, and > 12 M (Fig 1). Fecal sample were collected in 0.5 ml Brain Hearth Infusion broth (BHI) containing a cryoprotectant (15% glycerol), frozen within 2 hours and stored at -80°C until analysis. A written informed parental consent was obtained for each neonate before inclusion and the study was approved by the Saint Vincent de Paul research ethic Committee (ANR-07-PNRA-0028).

### *C*. *difficile* strains isolation and characterization

For specific *C*. *difficile* isolation and enumeration, fecal samples were serially diluted in peptoned water and were spread on *C*. *difficile* selective medium CLO-M (bioMerieux, Marcy l’Etoile, France) incubated for 24 h at 37°C under anaerobic conditions (CO_2_:H_2_:N_2_, 10:10:80, anaerobic chamber). *C*. *difficile* counts were expressed as Colony-Forming Units (CFU) per gram (wet weight) of feces (count threshold: 3.3 log_10_ CFU/g of feces). *C*. *difficile* isolates were identified using routine laboratory procedures and by PCR targeting the *C*. *difficile tpi* housekeeping gene [14]. *C*. *difficile* genes encoding TcdA and TcdB toxins were screened by PCR as previously described [14;15]. Qualitative determination of the *C*. *difficile* toxins TcdA and TcdB in fecal samples was performed using RIDASCREEN *Clostridium difficile* Toxin A/B enzymatic immunoassay (r-biopharm, Darmstadt, Germany) according to the manufacturer’s recommendations.

### PCR-ribotyping

Capillary gel-based electrophoresis PCR-ribotyping was performed as previously described by Bidet et al. [16]. After DNA amplification, 1 μL of a 1/200 dilution of each PCR product was mixed with 10.5 μL formamide and 0.5 μL GeneScan LIZ600 (Applied Biosystems, Foster City, USA) as an internal marker. After 30 s of denaturation at 90 °C, capillary electrophoresis was performed on 8-capillary 3500 Genetic Analyzer (Applied Biosystems). GeneMapper software (Thermo Fisher Scientific, Villebon-sur-Yvette, France) was used to analyze the banding patterns. PCR-ribotypes (RTs) were designated with either the standard nomenclature (European collection) (i.e. 032) when references were available or the specific nomenclature (i.e. FR082) of the French National Reference Laboratory for *C*. *difficile*.

### Multi-Locus Variable-number tandem-repeat Analysis (MLVA)

After DNA extraction, seven tandem repeat loci (A6, B7, E7, G8, C6, F3 and H9) were amplified by PCR. Briefly, three separate duplex and one singleplex PCR reactions were performed using the forward primers as follows: F3-FAM and A6-NED [17]; B7-FAM and E7-NED [17]; C6-FAM [18] and H9-NED [17]; G8-FAM [19]. Capillary-gel electrophoresis was performed with 1 μL of a 1/10 dilution of the PCR products. Raw allele data were acquired for each locus using GeneMapper software (Thermo Fisher Scientific). The distance between two strains was determined by calculating the STRD (summed tandem repeat differences). Isolates with an STRD ≤ 10 were defined as genetically related and clonal complexes were defined by an STRD ≤ 2. Genomic diversity was represented by the minimum spanning tree using the Manhattan coefficient (Bionumerics 5.1 software program).

### Statistical analysis

XLSTAT 2014 add-on software was used for statistical analysis. Fisher’s exact test was used for comparison of categorical variables. The Wilcoxon-Mann and Whitney U test for ranked data was used to compare continuous variable. A value of P < 0.05 (two-tailed) was considered significant. Univariate analysis was used to evaluate the relationship between *C*. *difficile* colonization and neonatal perinatal determinants documented such as sex, gestational age, birth weight, delivery mode, and maternal and PN antibiotic therapy.

## Results

### *C difficile* colonization

*C difficile* colonization of 121 PN was monitored up to 16 months. Characteristics of the PN included in this study are reported in Table 1. PN were born at a median gestational age of 32 W [interquartile, 29–33 W] and had a median birth weight of 1595 g [interquartile, 1200–1932 g]. No neonates suffered from necrotizing enterocolitis. Out of the 379 fecal samples analyzed, 199 (52%) from 97 neonates (80%) were *C*. *difficile* culture positive. During hospitalization, the frequency of *C*. *difficile* carriage increased from 20% at 1W to 61% at 1M (Fig 1). After HD, the proportion of carriage increased up to 74% and tended to decrease after 12 M (53%). Additionally, after HD, *C*. *difficile* colonization was significantly higher in PN less 1 year of age as compared to PN more than 1 year of age (88% vs 53%; P = 0.0002). The mean levels of *C*. *difficile* colonization decreased significantly (P = 0.027) over time from 6.0 log_10_ ±1.3 CFU/g of feces at 1 W to 5.2 log_10_ ±1.0 CFU/g for samples collected after 12 M (Fig 1).

**Table 1:** Characteristics of the premature neonates included in the study.

Number of neonates	121
Gestational age (weeks)^a^	31.2 ± 2.8
Birth weight (g)^a^	1568 ± 501
Birth mode (vaginal/c-section)	45/76
Gender (male/female)	49/72
Antibiotics (0–1 month) (days)^a^	4 ± 3 (min 1 –max 13)
Singleton pregnancy	61

^**a**^Mean ± SD

Of the 199 *C*. *difficile* strains, 10 (5%), isolated from 8 PN, were toxigenic. PCR screening showed that they were all *tcdA*^+^/*tcd*B^+^ except one *tcdA*^-^/*tcd*B^+^. Immunoassay qualitative determination for TcdA/TcdB showed the presence of the toxins in the corresponding PN samples. None of the toxigenic strains were positive for the binary toxin genes *cdtA* and *cdtB*. Toxigenic strains mean levels of colonization were 5.4 log_10_ ±1.0 CFU/g of feces and was comparable to the levels of non-toxigenic strains (5.3 log_10_ ±0.9) (P = 0.89).

### Distribution of PCR-ribotypes

A total of 13 different RTs were identified during the study (Fig 1). The non-toxigenic RTs 032 and FR082 were the most frequently isolated and accounted for 78% of all the strains (respectively 40% and 38%). During hospitalization, 95% of the strains isolated belonged to either FR082 (35%) or 032 (60%) RTs. After HD, a higher diversity in RTs was observed although FR082 (40%) or 032 (28%) remained predominant (Fig 1). Concerning the ten toxigenic strains isolated from 8 PN during the post-hospitalization period, they belonged to the RTs 014/020/077 (n = 2), 017 (n = 1), 106 (n = 1), 126 (n = 3), or 430 (n = 3) (Fig 1). One PN was colonized by the toxigenic RT 126 that was consecutively recovered at 1-3M, 3-6M, and 6-9M sampling times. For the other PN, the toxigenic RTs were recovered only once (at 6-9M (RT 106 (n = 1)), or 9-12M (RT 017 (n = 1)), or after 12M (RTs 430 (n = 3) and 014/020/077 (n = 2)).

In terms of follow-up, only four PN showed an absence of *C*. *difficile* colonization during the whole study period. For 53 PN (44%), at least one stool sample during both hospitalization and post-hospitalization periods were collected. This allowed to identify six *C*. *difficile* patterns of colonization with an important inter-individual variability (Table 2).

**Table 2:** *C*. *difficile* ribotype colonization patterns among the preterm neonates during the hospitalization and post-hospitalization periods.

Profiles^a^ (n)	Ribotype patterns		Neonates^b^ (n)
	Hospital	Post-hospital	
A (14)	ND^c^	032	4
		FR082	2
		140	2
		126*	1
		010 shifted to 140	1
		FR082 shifted to 032	2
		FR082 shifted to 014/020/077*	1
		FR082 shifted to 430*	1
B (19)	FR082	ND	1
		FR082	10
		010	1
		031	1
		FR082 shifted to 032	1
		FR082 shifted to 430*	2
		FR082 shifted to 010	1
		FR082 shifted to 031	1
		FR082 shift to 010 shift to 009	1
C (14)	032	032	7
		FR082	1
		032 shifted to 140	1
		032 shifted to 031	2
		032 shifted to FR124	1
		FR082 shifted to 017*	1
		FR082 shifted to 032	1
D (1)	140 shifted to 032	140	1
E (1)	FR082 shifted to 032	FR082	1
F (4)	ND	ND	4

^a^Profiles are based on the colonization pattern during hospitalization and changes observed during post-hospitalization periods

^**b**^ Preterm neonates with at least 1 Hospital and 1 Post-hospital sample analyzed

^**c**^ ND, not detected (<3.3 log_10_ CFU/g feces)

*, Toxigenic strain

### Multi-Locus Variable-number tandem-repeat Analysis (MLVA)

**Fig 2:**
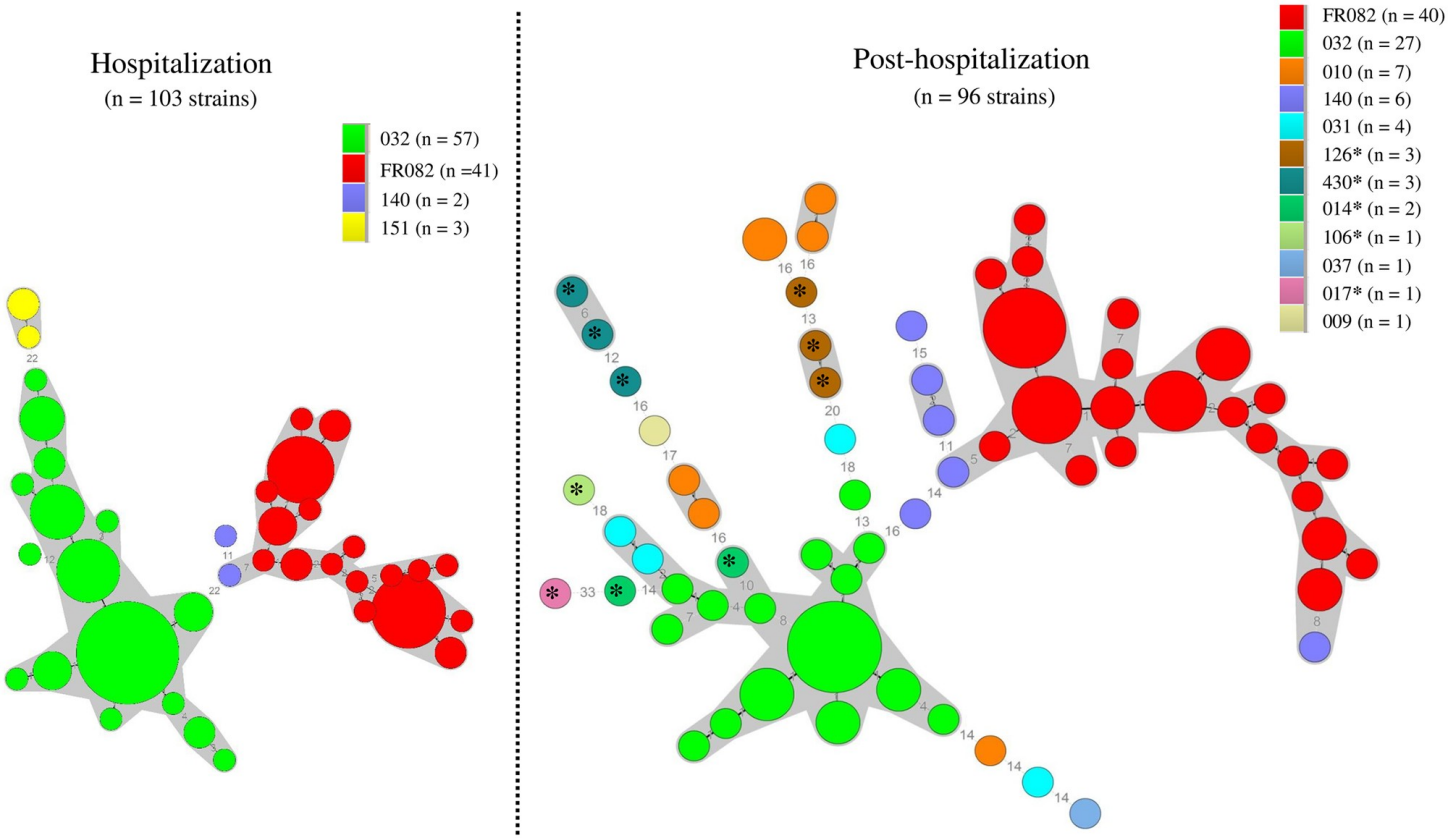
Minimum-spanning tree representation of Multi-Locus Variable-number tandem-repeat Analysis (MLVA) data of the 199 *C*. *difficile* isolates. The circles represent unique MLVA types and are scaled to member count. The numbers between the circles represent the STRDs (summed tandem repeat differences) between MLVA-types. Grey areas represent genetically related MLVA types (STRD ≤10). MLVA-types linked by a plain line are part of the same clonal complex (STRD ≤2). PCR-ribotypes are color-coded; * toxigenic strains.

MLVA was used to investigate the genetic relationship among *C*. *difficile* strains. During the hospitalization period, MLVA distinguished respectively 16 and 18 MLVA-types of RTs 032 (n = 57) and FR082 (n = 41). Within RT 032, all strains but one were genetically related (STRD ≤ 10), and 53 strains formed a clonal complex (STRD ≤ 2); FR082 strains were all genetically related and formed two clonal complexes of 38 and 3 strains respectively (Fig 2). After HD, *C*. *difficile* RTs 032 (n = 27) and FR082 (n = 40) still formed clusters including virtually all MLVA-types, most of them being in the same clonal complex (Fig 2). Concomitantly, infants were colonized with different RTs showing a higher diversity in their MLVA-types. Concerning toxigenic RTs, *C*. *difficile* strains were characterized by the absence of genetic relationship with the non-toxigenic strains as shown by their distinct MLVA-types, except for one 014 stain included in the 032 cluster (Fig 2).

### Perinatal determinants and *C*. *difficile* colonization

PN clinical data available were gestational age, birth weight, delivery mode, maternal and PN antibiotic therapy. At 1W, a higher gestational age (P = 0.006) and a higher birth weight (P = 0.016) were the only perinatal determinants significantly associated with higher *C*. *difficile* colonization frequency. No statistical differences were observed between the levels of *C*. *difficile* colonization and the different perinatal determinants.

## Discussion

In the present study, we investigated the dynamics of carriage and colonization by *C*. *difficile* in 121 PN first hospitalized in the same NICU. Early after birth, we showed a high colonization rate with a very limited number of genetically related non-toxigenic *C*. *difficile* strains. After hospital discharge, PN acquired a higher diversity of non-toxigenic strains and carriage of toxigenic strains emerged.

Numerous studies have reported *C*. *difficile* colonization in neonates, infants and children (for review see [10]), but none was specifically performed on PN. Therefore, comparison of our results with available data in the literature was challenging. Studies from the 1980s in full-term neonates showed that colonization by *C*. *difficile* increased during the first month of life, peaked around 6 months and declined afterwards [20;21]. Lees et al reported a *C*. *difficile* colonization rate of 20–30% in neonates less than 1 month of age, 10–25% in infants of 1 month to 1 year of age and 5–10% in children more than 1 year of age, although a large inter-study variation exists [10]. In our study, the average frequency of colonization is higher than previously reported in neonates [11;13;22]. This is in accordance with studies that reported recovery rates from neonates in NICU higher than those from neonates in regular nurseries, likely due to longer stays in NICU favoring colonization from the hospital environment [11;13;22]. Besides, after HD, *C*. *difficile* colonization was significantly higher in PN less than 1 year of age as compared to PN more than 1 year of age. This rate is higher than previously reported in neonates [11;22;23].

In neonates, *C*. *difficile* colonization has been proposed to be related to different perinatal determinants such age, type of delivery, feeding, and intestinal microbiota [11;24;25]. In PN, NICU environment and antibiotic courses has been shown to influence clostridia colonization [13]. In the current study, at 1W, a higher carriage frequency of *C*. *difficile* was observed in PN with a higher gestational age and birth weight. One explanation can be the fact that medical care of PN with lower gestational age and birth weight include a limited environmental exposure in the nursery that could delay the time course of *C*. *difficile* acquisition.

Among the 199 strains isolated, 95% were non toxigenic and belonged to only two RTs (FR082 and 032). These two RTs were carried by 77% of the PN during the study period. During the hospitalization period, we showed the persistence of both RTs FR082 and 032 over time in a transient state. This is consistent with a possible cross transmission within the care unit as it has been previously reported for full term neonates [26;27]. *C*. *difficile* variation with time has been reported for adults [28]. From hospital discharge onwards, although FR082 and 032 were still the most frequent RTs, colonization regularly increased in diversity, likely due to a diversification of environmental exposure sources. Simultaneously, the mean levels of *C*. *difficile* colonization decreased significantly over time. Through this one-year longitudinal study, our data show that PN carry or are colonized by dominant or subdominant *C*. *difficile* strains either transiently or over a long-term period. In terms of strains relatedness, MLVA typing clearly showed that strains within FR082 and 032 RTs belonged either to the same clonal complex or were genetically related independently of the isolation period. The identification of clonal complexes by MLVA strongly suggests a potential hospital transmission. The predominance of these two non-toxigenic clones raises the question as to whether they are specifically adapted to PN.

In our study, only 10 (5%) of the *C*. *difficile* isolates were toxigenic. This frequency is lower than the 20 to 71% previously reported in infants [11;29]. In our PN population, the toxigenic strains belonged to the RTs commonly isolated from infants [23;29;30], adults with *C*. *difficile* infections (014/020/077, 017, 106, and 126) [4], water, and animals (126, 430) [31]. The absence of *C*. *difficile* infection in children colonized by toxigenic strains is still a matter of debate. It could be due to the absence of toxin receptors, or to their inability to bind to their receptors [10;11]. Gut microbiota has also been proposed to confer a protective barrier effect against *C*. *difficile* overgrowth in neonates and infants [10;11;25]. In our study, all toxigenic strains were identified only after hospital discharge. Possible explanations include PN specific host factors, and the occupation of a specific intestinal ecological niche by the predominant FR082 and 032 RTs that result in exclusion of other *C*. *difficile* strains including toxigenic ones.

Limitations of our analysis include data from a monocentric study. Therefore we cannot rule out the possible over-representation of both FR082 and 032 RTs in the care unit. A multi-centric study would help understand whether these two non-toxigenic clones may be or not specifically adapted to PN. The specimens used in our study were collected 10 years ago, and epidemiology may have changed over time. However, face with the absence of available data, our study provides valuable information concerning the pattern of colonization by *C*. *difficile* over a one-year period in PN, an understudied population at high risk of colonization by this species.

In conclusion, the dynamics of *C*. *difficile* colonization in PN followed a specific pattern. Throughout the hospitalization period, PN showed an increasing frequency of colonization by non-toxigenic *C*. *difficile* strains. After HD, diversification of RTs colonization and colonization of toxigenic strains occurred rapidly. Altogether, these data reflect an acquisition of *C*. *difficile* pathogenic strains circulating from the community environment, PN representing afterwards a transient reservoir.
